# The anisotropic broadband surface plasmon polariton and hot carrier properties of borophene monolayer

**DOI:** 10.1515/nanoph-2021-0599

**Published:** 2022-01-11

**Authors:** Chaochao Jian, Xiangchao Ma, Jianqi Zhang, Jiali Jiang

**Affiliations:** School of Physics and Optoelectronic Engineering, Xidian University, Xi’an 710071, China

**Keywords:** borophene monolayer, first-principles, hot carriers, surface plasmon polariton

## Abstract

Borophene monolayer with its intrinsic metallic and anisotropic band structures exhibits extraordinary electronic, optical, and transport properties. Especially, the high density of Dirac electrons enables promising applications for building low-loss broadband SPP devices. However, a systematic characterization of the surface plasmon polariton (SPP) properties and hot carriers generated from the inevitable SPP decay in borophene has not been reported so far. Most importantly, the mechanism for SPP losses remains obscurely quantified. In this work, from a fully first-principles perspective, we explicitly evaluate the main loss effects of SPP in borophene, including the Drude resistance, phonon-assisted intraband and direct interband electronic transitions. With this knowledge, we further calculate the frequency- and polarization-dependent SPP response of borophene, and evaluate some typical application-dependent figure of merits of SPP. On the other hand, we evaluate the generation and transport properties of plasmon-driven hot carriers in borophene, involving energy- and momentum-dependent carrier lifetimes and mean free paths, which provide deeper insight toward the transport of hot carriers at the nanoscale. These results indicate that borophene has promising applications in next-generation low-loss optoelectronic devices and photocatalytic reactors.

## 1 Introduction

Surface plasmon polariton (SPP) [1] is the collective oscillation of free electrons at metal/dielectric interfaces induced by incident light with a specific frequency, which has been attracting great attention in the realm of nanophotonics and optoelectronics. In these systems, the electromagnetic field of SPP is evanescently confined in the normal direction of the interface, thus breaking the optical diffraction limit [2]. These unique properties enable the miniaturization of optoelectronic devices and nanoscale regulation of light field, and therefore have broad applications, such as integrated nanophotonics [3], photocatalysis [4], and sensing detection [5].

Practically, it is required that SPP exhibits low damping and wide visible plasmonic response, which is one of the current research hotspots in exploring the SPP properties of new materials [6, 7]. In the limit of extreme quantum confinement and low dimensionality, excellent SPP is expected to appear in emerging new materials. For example, Nao Zhang et al. indicated that the SPP propagation length of Ag nanowire is prominently increased to about 7 μm [8]. Meanwhile, two-dimensional (2D) materials are widely studied, especially the well-known graphene [9] and noble metal films (Au and Ag) [10]. However, the frequencies of plasmonic response in these materials are mostly limited to the infrared region, where the development of optoelectronic detectors or light sources is relatively slow compared with the visible light range. Although the frequencies of plasmonic response can usually be extended into the visible region by increasing the carrier concentration, it also adversely results in high loss due to the increase of phase space for electron–phonon scattering. Therefore, it is urgent to find a plasmonic material with low losses and broadband response for further development applications in optoelectronics, nanophotonics, and photocatalysis.

Recently, some theoretical studies have predicted that borophene, a single atomic-layer of boron, shows unique electronic, optical, and transport properties, which is related to the anisotropy of the crystal structure. For instance, Yuefei Huang et al. predicted that the plasmon frequency of borophene reaches the near-infrared and even visible regions, notably with no necessity of doping [11]. Moreover, Chao Lian et al. indicated that collective excitations in borophene exhibit two major plasmon modes with low damping rates extending to the ultraviolet region [12]. Although there is growing interest in understanding and characterizing the excellent SPP properties of borophene monolayer, the important scattering effects that determine the losses of SPP in the previous theoretical calculations remain obscurely quantified. For example, the value of momentum relaxation times characterizing the electron scattering effects is generally replaced by an empirical parameter or extrapolated values [13, 14], which greatly limits the accuracy of results.

In this paper, based on the first-principles calculations without any empirical parameters, we explicitly calculate the Drude momentum relaxation times and frequency-dependent momentum relaxation times of phonon-assisted intraband electronic transitions, respectively. Based on these estimated momentum relaxation times, our calculated optical responses of bulk Ag are in good agreement with experimental results, indicating the reliability of our theoretical calculations. Furthermore, we systematically investigate frequency- and polarization-dependent SPP responses of borophene monolayer, and evaluate the figure of merits of SPP for typical plasmonic applications. On the other hand, the inevitable decay of SPP through Landau damping generally results in hot carriers, which have been proven to exhibit application potential in energy conversion [15], photocatalysis [16], and photodetection [17], is also a recent research hotspot. Therefore, we also investigate the distributions in the energy and momentum spaces, and transport properties of the plasmon-driven hot carriers in borophene monolayer.

Overall, this work shows that borophene monolayer exhibits excellent surface plasmon polariton (SPP) and novel plasmon-driven hot carrier properties. Specifically, we find that borophene monolayer shows very distinct conducting properties along the two lattice directions. As a result, the SPP of borophene monolayer is very sensitive to the polarization of incident light. In particular, very low losses of SPP are obtained along with both lattice directions, and the low-loss SPP along one of the two lattice directions extends well into the visible region. In addition, it is mainly high-energy hot holes that are generated from the decay of SPP. Meanwhile, the hot carriers exhibit highly anisotropic distributions in momentum space. The transport results show that the lifetime and mean free path of hot carriers reach 10 fs and 3 nm, respectively, which is comparable with that of Au–Al intermetallic compounds. These results provide crucial guidelines for designing high-efficiency optoelectronic devices based on borophene monolayer.

## 2 Methods

**Computational details**: Firstly, the electronic structure, phonon spectrum, and electron–phonon matrix elements are calculated from first principles using density functional theory (DFT) as implemented by the open-source code JDFTx software [18]. We use full-relativistic norm-conserving pseudopotentials, the generalized gradient approximation functional of Perdew, Burke, and Ernzerh for describing the exchange-correlation energy and truncated Coulomb interactions to isolate periodic images for the 2D systems [19]. The plane-wave cutoff energy is 25 Hartrees, and Fermi–Dirac smearing is 0.01 Hartrees. A 14 × 8 × 1 *k*-point mesh in the Brillouin zone of borophene monolayer is used during structural and electronic properties calculations, and a dense 42 × 24 × 1 *k*-point mesh is used for the accurate density of states calculations. Phonon calculations employ a 7 × 4 × 1 supercell for borophene monolayer. All the parameters above have been tested to give well-converged results. Next, all electron, phonon, and electron–phonon matrix elements obtained through DFT are respectively converted to maximally-localized Wannier function (MLWF) basis representation [20, 21] and then interpolated to extremely fine electron wave-vector **k** and phonon wave-vector **q** meshes. Specifically, we use 46 Wannier bands for borophene monolayer to exactly reproduce the orbital energies, phonon energies, and electron–phonon matrix elements up to at least 30 eV above the Fermi level, which is sufficient for fully converging the sum over states in the second-order perturbation theory. Finally, we systematically evaluate the optical response and transport properties of borophene monolayer by Monte Carlo Brillouin-zone integration.

**Optical responses**: Fundamentally, it is mainly the interband and phonon-assisted intraband electronic transitions, and Drude resistance that determine the optical response of a metallic material [22]. In this work, we express the optical response of borophene monolayer by the frequency-dependent complex conductivity σ(ω) , of which the real part is calculated by [23, 24]:

(1) Reσ(ω)=σ0τD0-1⋅τD-1(ω)[τD-1(ω)]2+ω2+Reσinter(ω)

where the first term accounts for the effects of phonon-assisted intraband electronic transitions and Drude resistance, and the second term represents the effect of direct interband electronic transitions.

For the effect of phonon-assisted intraband electronic transitions in the first term, we calculate the frequency-dependent momentum relaxation rates $\tau_{D}^{-1}$ from the Eliashberg spectral function [25, 26]. Note that the general extrapolation methods in calculating the contribution of phonon-assisted electronic transitions to optical response suffer from the defects of singularity [27, 28].

(2) $$\tau_{D}^{-1}\left(\omega \right)=\frac{2\pi }{\hbar \text{g}\left(\epsilon_{F}\right)b_{T}\left(\hbar \omega \right)}\sum_{\alpha }\int_{BZ}\frac{dq}{{\left(2\pi \right)}^{d}}G_{q\alpha }^{p}b_{T}\left(\hbar \omega -\hbar \omega_{q\alpha }\right)$$

where ${\text{b}}_{T}\left(\epsilon \right)\equiv \frac{\epsilon }{1-e^{-\frac{\epsilon }{k_{B}T}}}$ and the dimensionless $G_{q\alpha }^{p}$ is defined as:

$$G_{q\alpha }^{p}\equiv \sum_{nn^{\prime }}\int_{BZ}\frac{g_{s}\Omega dk}{{\left(2\pi \right)}^{d}}{\left|g_{\left(k+q\right)n^{\prime },kn}^{q\alpha }\right|}^{2}\left(1-\frac{v_{kn}\cdot v_{\left(k+q\right)n^{\prime }}}{\left|v_{kn}\right|\left|v_{\left(k+q\right)n^{\prime }}\right|}\right)\times \delta \left(\epsilon_{kn}-\epsilon_{F}\right)\delta \left(\epsilon_{\left(k+q\right)n^{\prime }}-\epsilon_{F}\right)$$

where $\epsilon_{kn}$ is the energy of electron with wave-vector k in band *n*, and $\hbar \omega_{q\alpha }$ is the energy of phonon with wave-vector q in mode α ; $\epsilon_{F}$ is the Fermi energy and $g\left(\epsilon_{F}\right)$ is the density of electronic states near the Fermi level; $g_{\left(k+q\right)n^{\prime },kn}^{q\alpha }$ is the electron–phonon matrix element with electronic states labeled by wave-vector k , q , and band indices n , $n^{\prime }$ ; Ω is the unit cell volume; $v_{kn}$ is the band velocity with wave-vector k and band *n*; $k_{B}$ is Boltzmann constant, and T represents the standard temperature ( T=298 K ); $g_{s}=2$ is the spin-degeneracy factor, and d is the dimension, which is 2 for 2D materials.

For the Drude resistance effect in the first term, the ratio of DC conductivity to the average Drude momentum relaxation time is calculated by: [23]

(3) $$\frac{\sigma_{0}}{\tau_{D0}}=\int_{BZ}\frac{e^{2}g_{s}dk}{{\left(2\pi \right)}^{d}}\sum_{n}\delta \left(\epsilon_{kn}-\epsilon_{F}\right)\left(v_{kn}⊗v_{kn}\right)$$

For the second term, the real part of conductivity due to direct interband transitions is calculated by [29]:

(4) Reσinter(ω)=ϵ0⋅ω⋅(πe2ω2∫BZgsdk(2π)d∑nn′(fkn−fkn′)δ(ϵkn′−ϵkn−ℏω)(vknn′∗⊗vknn′))

where $f_{kn}$ is Fermi occupation (with *k*_B_*T* ∼ 0.00094 Hartrees) of electrons with wave-vector k in band *n*; $v_{knn^{\prime }}$ is the matrix-elements of the velocity operator; $\epsilon_{\text{0}}$ is the vacuum dielectric constant. Finally, the Brillouin-zone integration is carried out using Monte Carlo sampling with ∼10^6^ k points, with all terms in the integrand calculated efficiently using the Wannier representation as discussed above.

**Transport properties**. Considering the effects of electron–electron and electron–phonon scattering, we discuss the transport properties of hot carriers generated by the inevitable decay of SPP in borophene monolayer. For the electron–electron scattering contribution, the imaginary part of the quasiparticle self-energy is calculated by [22]:

(5) Im∑kne−e=∫BZdk′(2π)d∑n′∑GG′ρ˜knk′n′(G)ρ˜knk′n′∗(G′)×4πe2|k′−k+G|2Im[ϵGG′−1(k′−k,ϵkn−ϵk′n′)]

where ${\tilde{\rho }}_{knk^{\prime }n^{\prime }}\left(G\right)$ are density matrices expressed in the plane-wave basis, and $\epsilon_{GG^{\prime }}^{-1}$ is the RPA dielectric matrix for reciprocal lattice vectors G and $G^{\prime }$ .

For the electron–phonon scattering contribution, the imaginary part of the lowest order electron–phonon self-energy is calculated by [30, 31]:

(6) Im∑kne-ph=∑n′α∫BZdk′(2π)d|g(k+q)n′,knqα|2Im[nq,α+1−fkn′ϵkn−ϵk′n′−ℏωq,α−iη +nq,α+fkn′ϵkn−ϵk′n′+ℏωq,α−iη]

where η is a small Lorentzian broadening considering the effect of thermal oscillation ( η = 25 meV); $n_{q,\alpha }$ is the Bose occupation of phonon state with wave vector q ( $q=k^{\prime }-k$ ) and polarization index α, and the other terms are the same as that in Eq. (2). Correspondingly, the relaxation lifetime including electron–electron scattering and electron–phonon scattering is calculated by τkn=ℏ2(Im∑kne-ph+Im∑kne-e) , and the mean free path is calculated by $\lambda_{kn}=\nu_{kn}\cdot \tau_{kn}$ , where $\tau_{kn}$ and $\nu_{kn}$ are lifetime and group velocity of the carrier in the electronic state kn , respectively.

## 3 Results and discussion

### 3.1 Structure and stability

**Figure 1:**
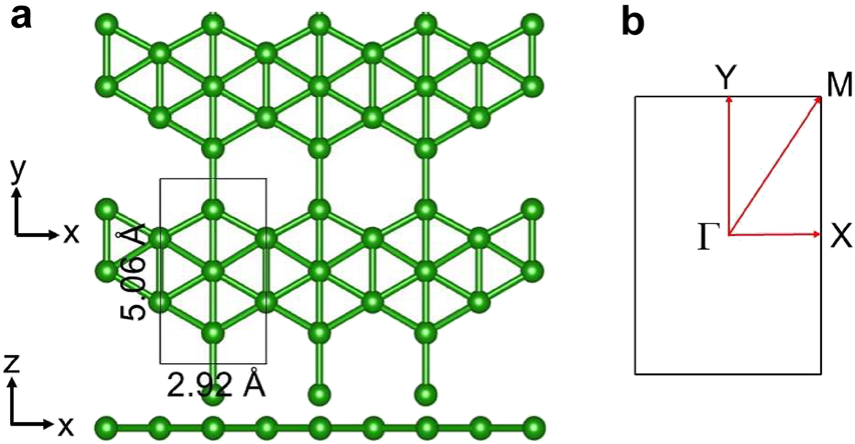
Optimized lattice structures of borophene monolayer. (a) Top and side views of the optimized lattice structures of borophene monolayer. The area enclosed by a black rectangle corresponds to a unit cell of borophene. (b) The first Brillouin zone of borophene monolayer, the irreducible ***k***-point path Γ-*X*-M-*Y*-Γ-M is used for calculating its band structure and phonon spectrum.

**Figure 2:**
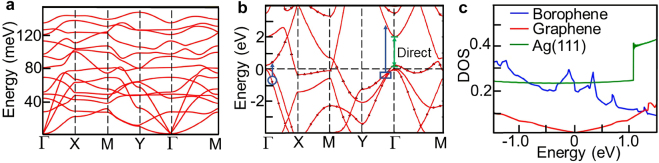
Phonon dispersions and band structures of borophene monolayer. (a) Phonon dispersion and (b) band structure of borophene monolayer. The black scatters represent band structures calculated by DFT calculations, and the red lines are obtained through interpolation of MLWFs. The green double arrow in (b) indicates the direct band gap of about 2 eV at Γ point. (c) Density of states (DOS) of borophene, graphene, and Ag(111) monolayers (with a thickness of *t*_2D_ ≈ 2.36 Å) with the unit of states/eV/atom. The Fermi levels in (b) and (c) are set to 0 eV.

The optimized lattice structure of borophene monolayer $\beta_{12}$ is shown in Figure 1(a). Structurally, the unit cell of borophene is rectangular consisting of five atoms. Borophene has a 47-*Pmmm* space group with lattices constants of *a* = 2.92 Å and *b* = 5.06 Å, respectively, which are in good agreement with the reported experimental values [32]. Experimentally, the most stable $\beta_{12}$ borophene has been synthesized on Ag substrate [33, 34]. Moreover, the phonon dispersion of borophene monolayer in Figure 2(a) shows 15 phonon branches (3 acoustic, 12 optical) with a quite high optical phonon cut-off frequency (∼148 meV), which is comparable with the value of 200 meV in graphene. This indicates that the bonding among boron atoms in borophene monolayer is almost as strong as carbon–carbon bonds in graphene. Most importantly, the eigenvalues show that all the phonon modes have no imaginary frequencies, evidencing the dynamic stability of borophene monolayer.

Figure 2(b) shows the DFT calculated band structure in the first Brillouin zone, which is in excellent agreement with previous calculations [35]. Interestingly, along the Γ-*X* direction, the energy band crosses the Fermi level, indicating metallic properties of borophene. However, along the Γ-*Y* direction, there is a direct band gap of about 2 eV. As a result, borophene monolayer is a highly anisotropic metal, which has a great impact on its optical and carrier transport properties, which will be discussed in detail in the following sections. Examining the density of states (DOS) of borophene, graphene, and Ag(111) monolayers in Figure 2(c), we find that there is a relatively large DOS near the Fermi level in borophene monolayer, indicating high intrinsic carrier density, which is comparable with that of Ag(111) monolayers. Naturally, 2D materials with high intrinsic carrier density may sustain plasmon in the visible range that is particularly desirable for optical devices.

### 3.2 Surface plasmon polariton (SPP) properties

The optical response of plasmonic metallic materials across a broad frequency range, especially in the visible light region, is of great interest in many fields. Here, we focus on the SPP properties of borophene monolayer. For SPP, the decay channels include Landau damping and resistive dissipation. Generally, the Landau damping can be further divided into direct interband and phonon-assisted intraband electronic excitations. Physically, the relevant losses of SPP can be expressed by the imaginary part of the dielectric function, which is related to the real part of conductivity as

**Figure 3:**
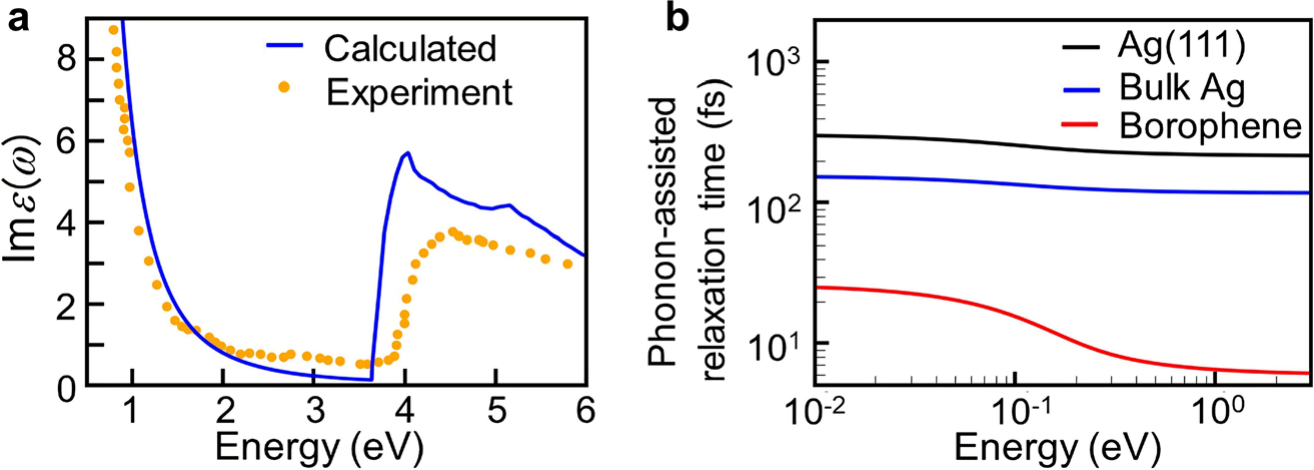
Frequency-dependent momentum relaxation times of Ag(111) monolayer, bulk Ag, and borophene monolayer. (a) Imaginary part of the dielectric function obtained by experimental measurement and our calculation, as a function of photon energy, in bulk Ag. (b) Momentum relaxation times of phonon-assisted intraband electronic excitations, as a function of photon energies, in Ag(111) monolayer, bulk Ag, and borophene monolayer.

**Figure 4:**
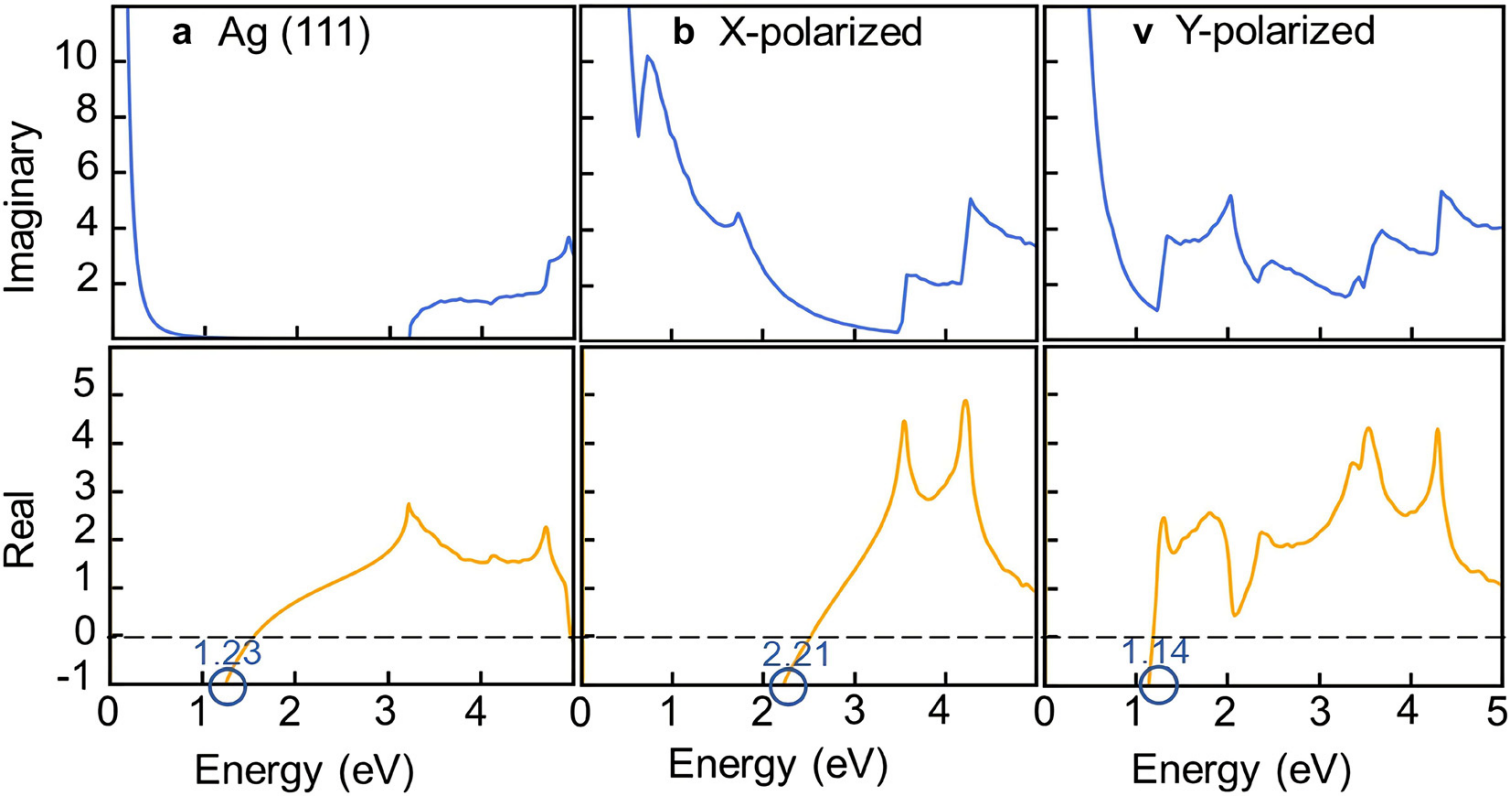
Imaginary and real parts of the complex dielectric function, as a function of photon energy, in Ag(111) monolayer (a) and borophene monolayer for X-polarized light (b) and Y-polarized light (c). The circles indicate the values of surface plasmon frequency $\omega_{sp}$ .

(7) Imϵ(ω)=Reσ(ω)ϵ0ω

where Reσ(ω) is the real part of frequency-dependent complex conductivity (Eq. (1)). Then we calculate the corresponding real part of the dielectric function using the Kramers–Kronig relation [36]. Firstly, to verify the accuracy of our theoretical methods, we calculate the imaginary part of the dielectric function of bulk Ag. As shown in Figure 3(a), our calculated results are very close to the experimental values [37], indicating that our theoretical methods can provide accurate descriptions of the microscopic decay mechanism of SPP for metallic materials. Note that a slight mismatch appears with increasing energy, which is caused by the inaccuracy of the PBE exchange-correlation approximation. Generally, the calculated band structures based on PBE exchange-correlation approximation tends to underestimate the interband excitation of a material. This explains the relatively small theoretical values of the imaginary part of dielectric function at about 3.7 eV with respect to the experimental values. On the other hand, the PBE functional also slightly underestimates the dispersion of band structures, which increases the electronic density of states and thus slightly increases the theoretical values of the imaginary part of the dielectric function relative to the experimental values. We plot in Figure 4 the calculated frequency-dependent optical dielectric function of Ag(111) and borophene monolayers across the entire infrared–ultraviolet range. Obviously, the plasmonic decay of borophene monolayer is higher than that of Ag monolayer in the energy range below 1 eV, where the plasmonic decay is mainly caused by the phonon-assisted intraband electronic excitations. Specifically, as shown in Figure 3(b), the phonon-assisted momentum relaxation time of borophene monolayer is much lower than that of Ag monolayer and drops dramatically over the 0.1–0.3 eV frequency range, originating from the existence of optical phonon with a maximum energy ∼0.15 eV in borophene monolayer. This indicates the larger plasmonic decay of borophene monolayer in the low-frequency range is related to the relatively strong scattering with optical-phonon. Moreover, the anisotropy of plasmonic decay in borophene monolayer is very apparent in comparing Imϵ for the polarization of incoming light (Figure 4(b) versus Figure 4(c)). The results indicate that at energies less than 1.6 eV, the plasmonic decay is higher for *X*-polarized light than for *Y*-polarized light and it is opposite above 1.6 eV, which originates mainly from the directionality of electronic excitations.

**Figure 5:**
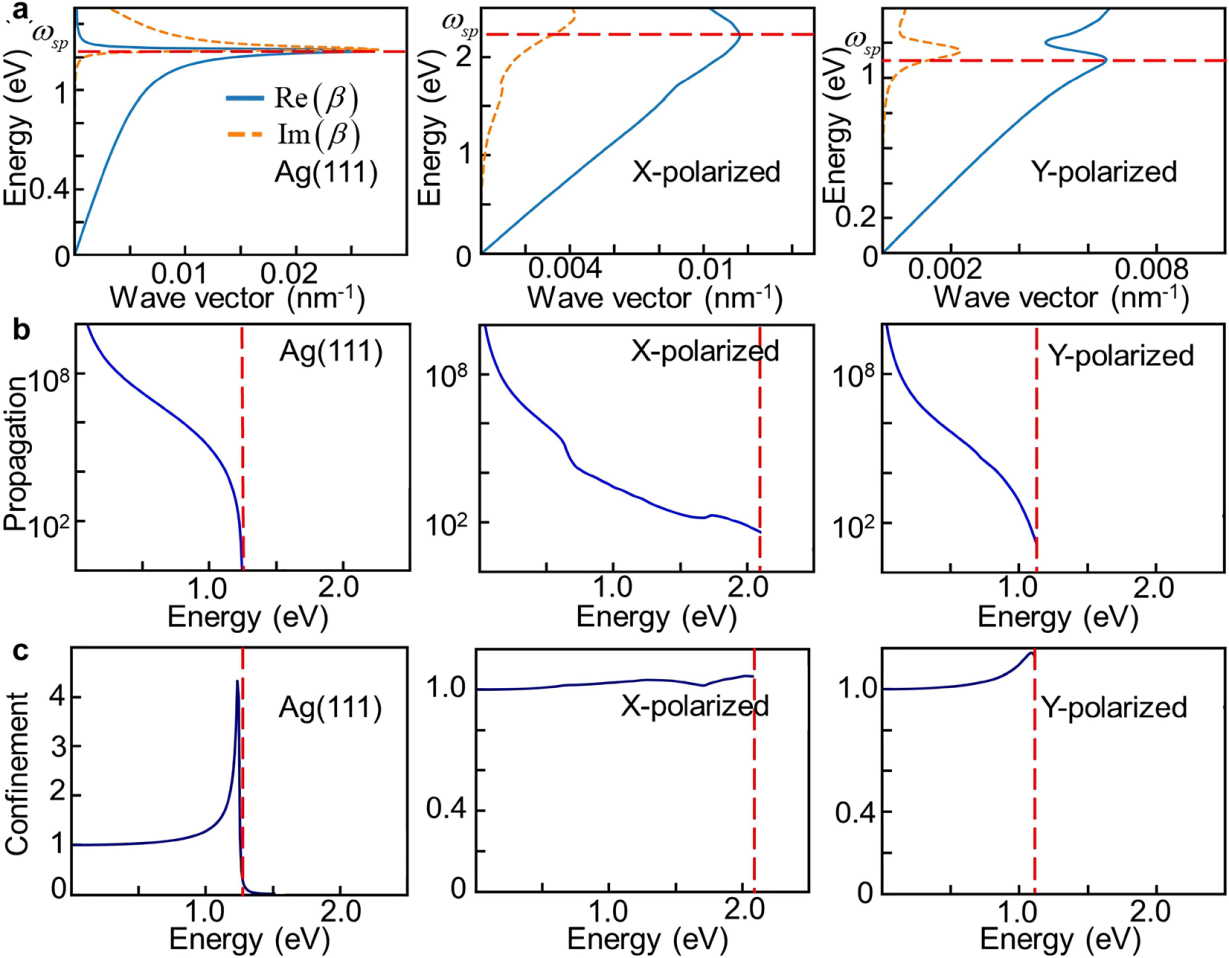
Plasmon dispersions and figures of merit of Ag(111) monolayer and borophene monolayer. (a) Plasmon dispersion. (b) Effective propagation length expressed with the ratio Re(β)Im(β) . (c) Confinement ratio $\frac{\lambda_{air}}{\lambda_{sp}}$ . The left panels indicate the results for Ag(111) monolayer, and the middle(right) panels for borophene monolayer for *X*-(*Y*-)polarized light. The red lines indicate the values of surface plasmon frequency $\omega_{sp}$ . The energy in (a)–(c) indicates the plasmonic response frequency. Obviously, the plasmonic response frequency of borophene monolayer for *X*-polarized light extends to the visible light range ( $\omega_{sp}$ = 2.21 eV).

Furthermore, we discuss the SPP dispersion properties of the metallic materials in a much broader energy range from infrared to visible light. SPP generally decays exponentially in the transverse direction, and the dispersion relation β of SPP can be expressed as [38, 39]:

(8) $$\beta =k_{0}\sqrt{\frac{\epsilon_{r}\epsilon \left(\omega \right)}{\epsilon_{r}+\epsilon \left(\omega \right)}}$$

where $k_{0}\equiv \frac{\omega }{c}$ is free space wave vector and c is the speed of light in vacuum. Note that throughout the paper, we use dielectric constant $\epsilon_{r}=1$ to define the air environment on top of borophene monolayer, and ϵ(ω) calculated by Eq. (7) describes the complex dielectric function of borophene monolayer. From the dispersion curves shown in Figure 5(a), the dispersions of SPP for *X*- and *Y*-polarized lights in borophene monolayer are completely different and strongly depend on the incident frequency. Practically, due to energy dissipation, the wave vector of SPP generally approaches a maximum, which is about 0.012 nm^−1^ (0.007 nm^−1^) for *X*-(*Y*-)polarized lights in borophene monolayer, and is slightly smaller than Ag monolayer. Particularly, SPP excited by X-polarized light expands the surface plasmon response frequency into visible light (∼2.2 eV).

For the application-dependent figures of merit of a given plasmonic material, we quantify the SPP effective propagation length with the ratio Re(β)Im(β) , which measures the SPP propagation length before an SPP loss most of its energy. The large values of the ratio indicate a low loss in plasmonic materials, which sensitively depend on frequency-dependent relaxation time. Meanwhile, we calculate the confinement ratio $\frac{\lambda_{air}}{\lambda_{sp}}$ ( $\lambda_{air}=\frac{2\pi c}{\omega }$ , λsp=2πRe(β) ), which reflects the degree of localization and depends mainly on macroscopic properties. As shown in Figure 5(b), in the entire plasmonic response range, the effective propagation length of borophene monolayer is comparable with that of Ag(111) monolayer. In addition, the effective propagation length of SPP for *X*-polarized lights is comparable with that for *Y*-polarized lights, but the SPP for *X*-polarized lights persists well into the visible region with moderate propagation length. In addition, Figure 5(c) shows that borophene monolayer exhibits confinement ratios close to 1 within the whole frequency range of plasmonic response, which is comparable to that of Ag(111) monolayer. Obviously, the confinement of SPP reaches the maximum values near the surface plasmon frequency, whereas the highest loss there results in the smallest propagation length. Significantly, the low-loss SPP of Ag(111) monolayer only exists within the infrared regime, but that of borophene monolayer extends to a much broader visible light region. This indicates that borophene monolayer is a better material for low-loss and broadband plasmonic response optoelectronic devices and photocatalytic reactors.

### 3.3 Hot carrier generation and distribution

**Figure 6:**
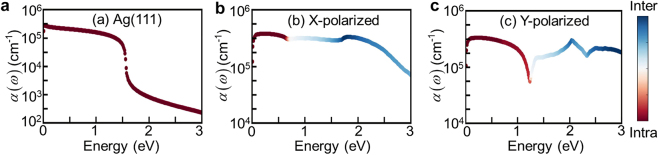
Comparison of direct interband and phonon-assisted intraband electronic transition contributions to hot carrier generations in Ag(111) monolayer (a) and borophene monolayer for *X*-polarized light (b) and *Y*-polarized light (c). The vertical axis indicates the optical absorption coefficients as a function of photon energy. The color scale incidents the relative contributions of direct interband (blue) and phonon-assisted intraband (red) electronic transitions for hot-carrier generations, which is defined as the ratio of the imaginary part of the dielectric function between the interband and phonon-assisted intraband electronic transitions.

Due to the well-known excellent light absorption ability of SPP, more efficient hot carriers (hot electrons and hot holes) generated by the non-radiative decay of the SPP are a sufficiently important application of metallic materials. According to the specific physical process of hot carrier generation, there are mainly two microscopic mechanisms: direct interband and phonon-assisted intraband electronic transitions. Hence, we systematically discuss the relative contributions of direct interband and phonon-assisted intraband electronic transitions to plasmon-driven hot carrier generations. Figure 6 shows the relative contribution of interband and intraband electronic transitions as a function of energy. As shown, in the energy range of 0–3 eV, hot carriers in borophene monolayer can be generated by both direct interband and phonon-assisted intraband electronic transitions, whereas hot carriers can only be generated by phonon-assisted electronic transitions in Ag(111) monolayer due to the large direct interband threshold of 3.2 eV. Moreover, Figure 6(b) and (c) indicate that the hot carrier generations are quite different for the two polarized lights, originating in the anisotropic electronic structure of borophene monolayer. The onset of direct interband transitions in borophene monolayer is at about 0.6 eV for *X*-polarized light and 1.3 eV for *Y*-polarized light, indicating that interband electronic transitions dominate the hot carrier generation in a wider energy regime for *X*-polarized light. It should be noted that the intraband thermal resistance, which competes with the phonon-assisted electronic transitions at very low frequencies, has a negligible effect on hot carrier generation.

On the other hand, we calculate the optical absorption coefficients from single-particle electronic excitations by [40]

(9) $$\alpha \left(\omega \right)=\sqrt{2}\frac{\omega }{c}{\left[\sqrt{\epsilon_{1}^{2}\left(\omega \right)+\epsilon_{2}^{2}\left(\omega \right)}-\epsilon_{1}\left(\omega \right)\right]}^{\frac{1}{2}}$$

where $\epsilon_{1}\left(\omega \right)$ and $\epsilon_{2}\left(\omega \right)$ are the real and imaginary parts of the dielectric function, respectively, which is obtained by Eq. (7). Although our theoretically predicted absorption coefficients of borophene monolayer shown in Figure 6(a) and (b) are up to 10^5^ in the entire visible region, the optical absorption of borophene monolayer is still less than 1% of the incident light, resulting in a very low photo-generated hot carrier generation efficiency. Our results agree well with the conclusion of Lyudmyla Adamska et al. [35] Therefore, it is necessary and valuable to focus on the hot carrier generation from the decay of SPP, which has efficient light absorption, for exploring the optoelectronic applications of borophene monolayer.

**Figure 7:**
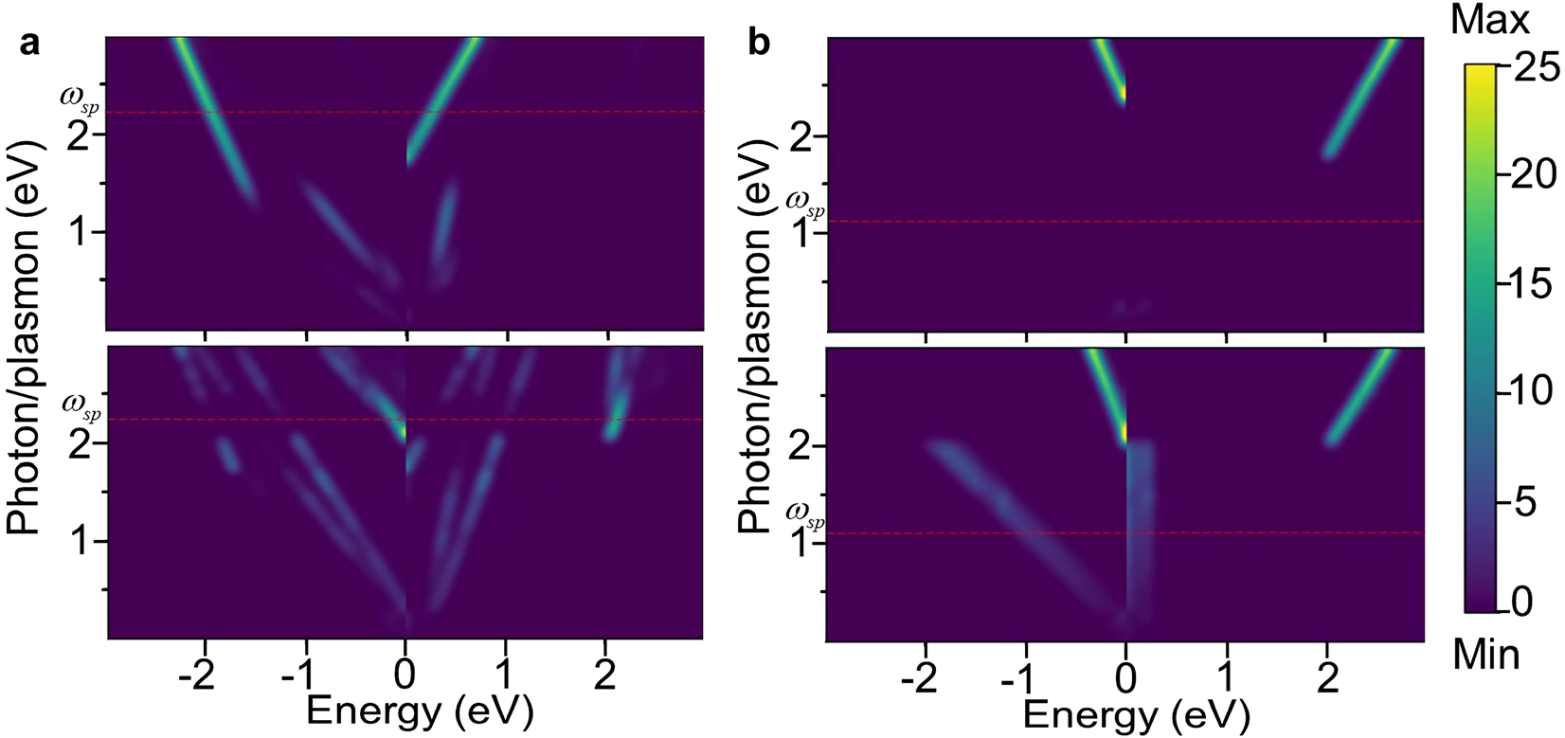
The energy distribution of hot carriers generated by direct interband (upper panels) and phonon-assisted intraband (lower panels) electronic transitions along with *X*-direction (a) and *Y*-direction (b) in the first Brillouin zone. The horizontal axis indicates the energy of photon/plasmon-generated carrier, and negative values for hot holes and positive for hot electrons. The red lines indicate the values of surface plasmon frequency $\omega_{sp}$ . The color bar indicates the distribution probabilities of hot carriers and the probability increases from purple to yellow. The Fermi energy is set to 0 eV.

Practically, it is important to know if the plasmon-driven hot carriers are high-energy electron-dominant or hole-dominant. Therefore, we focus on the energy distributions of the hot carriers. As shown in Figure 7, considering the direct interband electronic transitions, it is mainly high-energy holes with energies more than 1.5 eV that are generated along the *X*-direction, whereas high-energy electrons are mainly generated along the *Y*-direction. This can be understood from the electronic structures shown in Figure 2(b): along the Γ-*X* direction, the electrons below the Fermi energy marked by the circle move vertically upwards to the unoccupied band, resulting in the high-energy hot holes, and along the Γ-*Y* direction, the direct interband transitions of electrons within the marked rectangle generate the high-energy hot electrons. On the other hand, the phonon-assisted electronic transitions mainly produce high-energy hot electrons for excitation energies of more than 2 eV in both directions, whereas high-energy hot holes are mainly generated below 2 eV. Considering the plasmonic frequency range of borophene monolayer along *X* and *Y* directions, the energies of the plasmon-driven hot carriers will be smaller than the maxima of allowed SPP excitation energies, which are 2.21 and 1.14 eV for *X* and *Y* directions, repetitively. Therefore, it is mainly high-energy hot holes that can be generated for the decay of SPP through direct and indirect electronic transitions along the *X*-direction. For SPP along the *Y* direction, high-energy hot holes may also be generated by the decay of SPP through a relatively weak indirect electronic transition. Overall, it can be concluded that it is mainly high-energy hot holes that are generated from the decay of SPP in the entire plasmonic frequency range of borophene monolayer. This indicates that plasmon-driven hot carriers in borophene are mainly suitable for p-type collection/oxidation optoelectronics and photochemistry, which provides important guides for nanostructure design of borophene-based applications.

### 3.4 Hot carriers transport

**Figure 8:**
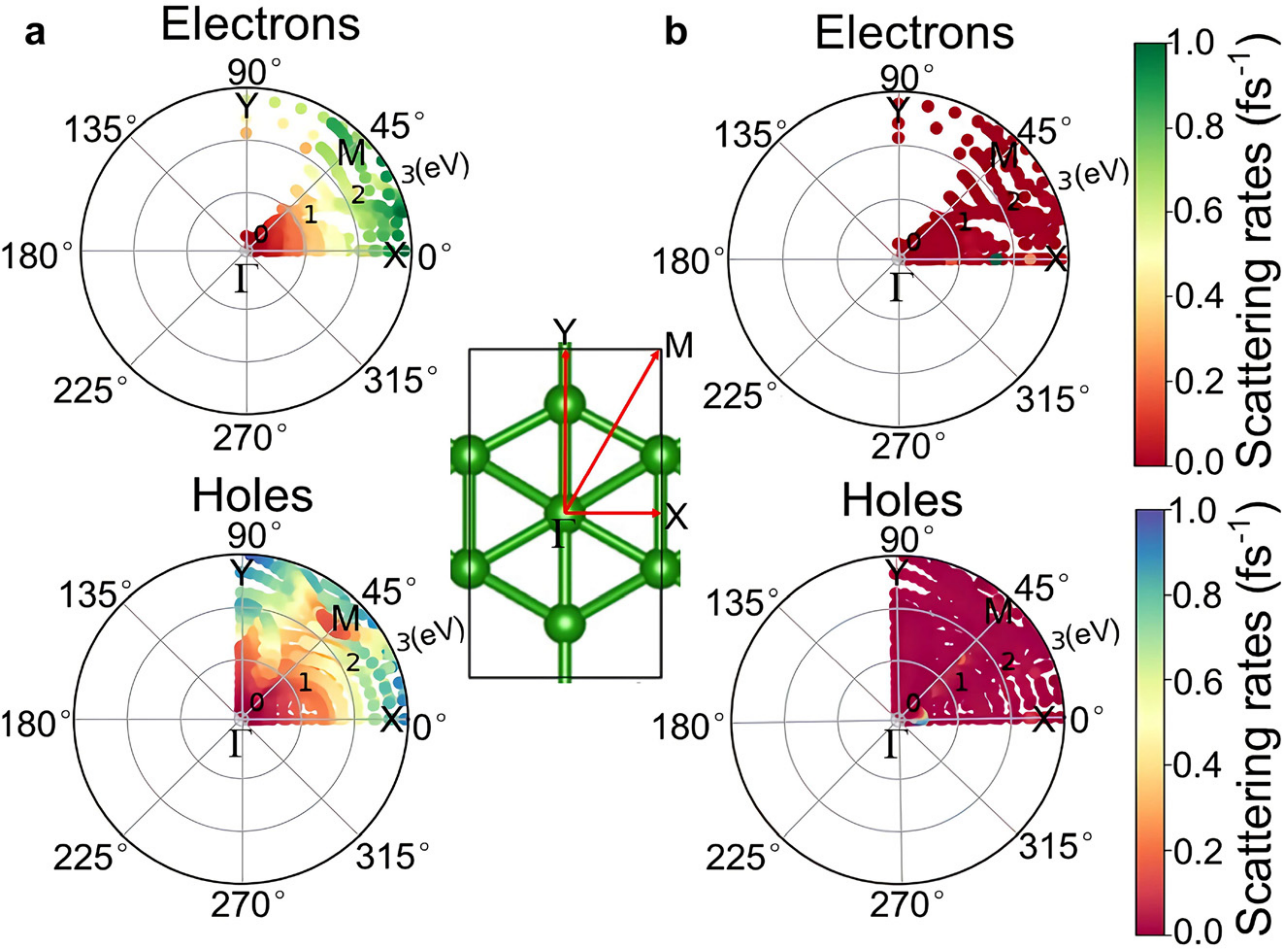
Electron–electron (a) and electron–phonon (b) scattering rates as a function of energy and momentum direction in borophene monolayer. The radial coordinates in each panel are the hot carrier energy relative to the Fermi energy, and the angular coordinates correspond to the hot carrier momentum directions. The color indicates the values of scattering rates.

The transport of photon/plasmon-driven hot carriers including their momentum-direction distributions [41] and mean free paths (MFP) is another critical parameter for instructing the preparation of high-performance optoelectronic devices. Figure 8 shows the calculated momentum-direction distributions of the photon/plasmon-driven hot carriers. Considering the crystal symmetry, only the results for the region with the angular coordinates ranging from 0° to 90° in the 2D momentum space of borophene monolayer are shown. It is worth noting that the momentum directions of the hot electrons are very uneven in the momentum space. As shown in Figure 8(a), the momentum directions of hot electrons are mainly in the region of angular coordinates near the *X* direction, whereas the momentum directions of hot holes are evenly distributed in the momentum space. These distinct transport directions of hot electrons and hot holes can realize the spatial separation of them, which is beneficial for decreasing their recombination. Moreover, the results show that hot carriers exhibit directional selectivity, which is of great theoretical guidance for hot carrier-based applications, such as direct photoelectric conversion on the surface of borophene monolayer or hot carrier injection into the contact semiconductor.

The lifetimes and mean free paths of hot carriers are calculated by fully considering both electron–electron and electron–phonon scattering processes. As expected for a metallic material conforming to Fermi-liquid theory [42], the electron–electron scattering rates shown in Figure 8(a) dominate far away from the Fermi energy due to a much larger phase space for such scattering events [43]. Meanwhile, electron–phonon scattering rates shown in Figure 8(b) play a major role near the Fermi energy, and the calculated values agree well with the results of Lyudmyla Adamska et al. [35] Moreover, the values of electron–phonon scattering rates in borophene monolayer, which are mainly distributed within about 0.5 fs^−1^, have the same order-of-magnitude as the electron–electron scattering rates. This indicates that electron–phonon scattering is sufficiently important compared with electron–electron scattering. Therefore, the effects of electron–phonon scattering on the hot carrier transport properties cannot be ignored, which is different from the general assumption that electron–phonon scattering occurs on a very slow time scale [42].

**Figure 9:**
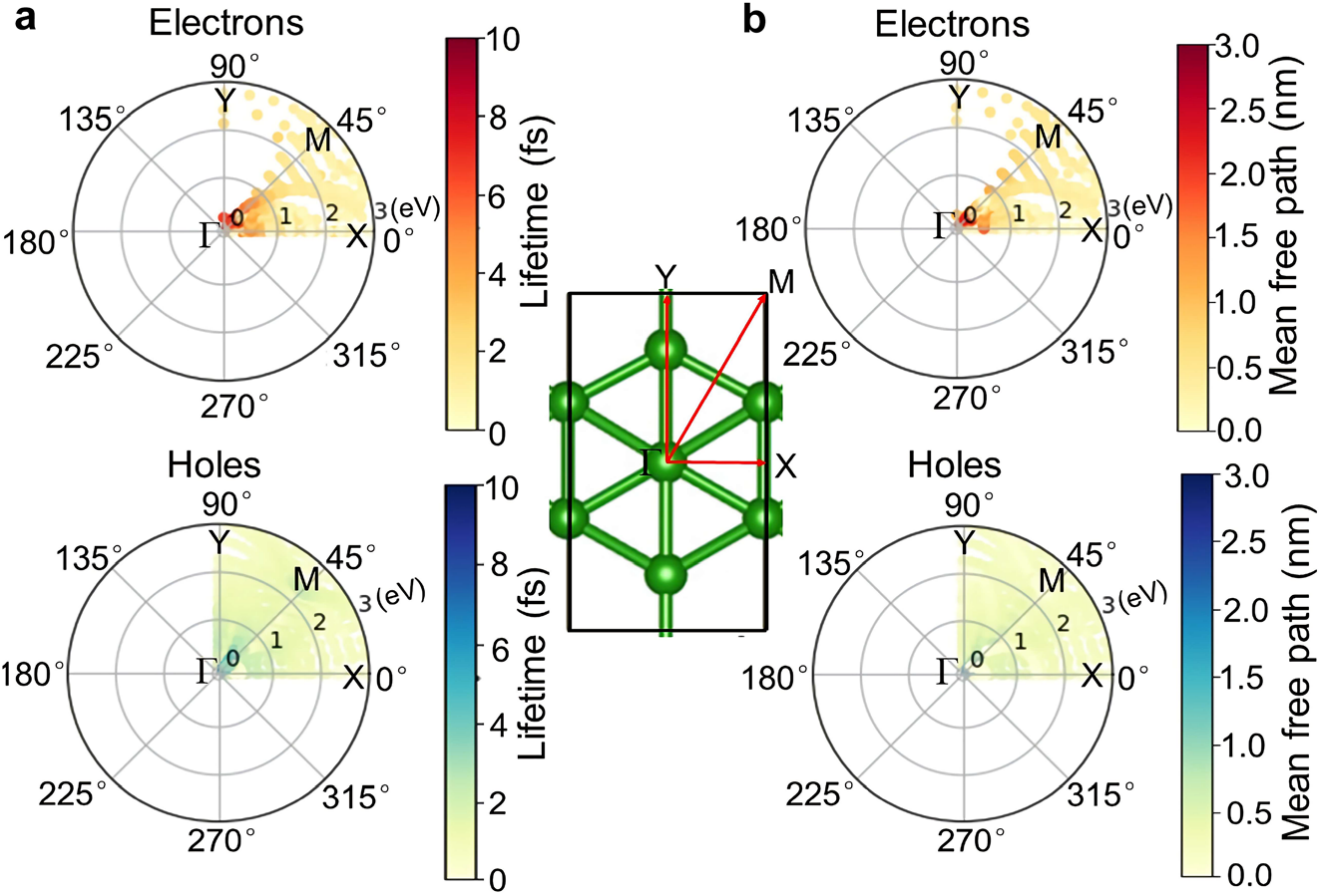
Lifetimes (a) and mean free paths (b) of photon/plasmon-driven hot carriers as a function of energy and momentum direction, accounting for both electron–electron and electron–phonon scattering contributions, in borophene monolayer. The radial coordinates in each panel are the hot carrier energy relative to the Fermi energy, and the angular coordinates correspond to the hot carrier momentum directions. The colors indicate the values of the lifetimes and mean free paths, respectively.

Figure 9 shows the calculated lifetimes and mean free paths as a function of the energy and momentum direction of hot carriers. Figure 9(a) shows that the hot carriers have the longest lifetimes of about 10 fs near the Fermi energy, and decrease rapidly away from the Fermi energy due to the increased electron–electron scattering, which is qualitatively similar to that of the noble metals (Au, Ag) [22]. Figure 9(b) shows that the mean free paths of hot carriers are within 3 nm, which approach that of Au–Al intermetallic compounds [44]. Notably, the lifetimes and mean free paths of hot electrons are almost the same as that of hot holes. Under momentum conservation, the momentum directions of hot carriers excited by *X*- and *Y*-polarized light are along the Γ-*X* and Γ-*Y* directions, respectively. As shown in Figures 8 and 9, for *X*-polarized light excitation, the lifetimes and mean free paths of low-energy hot electrons are higher than those of high-energy hot electrons, while *Y*-polarized light excitation can only generate high-energy hot electrons with smaller lifetimes and mean free paths. Meanwhile, hot holes excited by *X*- and *Y*-polarized light have similar lifetimes and mean free paths at the same energy. Overall, the novel transport properties of hot carriers derived from the structural anisotropy of borophene monolayer show the potential for directional transport of hot carriers.

## 4 Conclusions

Based on the first-principles calculations, we have systematically investigated the electronic structure, surface plasmon polariton (SPP), and plasmon-driven hot carrier properties of borophene monolayer. Our calculated phonon spectrum indicates that borophene, a single atomic-layer of boron, is dynamical stability in free-standing form. The unique electronic structures of borophene monolayer result in the highly metallic properties and anisotropy of electronic and optical properties. Next, the plasmon dispersions illustrate that the SPP of borophene monolayer is very sensitive to the polarization of incident light. And the effective propagation length of SPP is comparable with that of Ag monolayer, indicating low losses of SPP in borophene monolayer. In particular, the low losses SPP of borophene monolayer under the *X*-polarized incident light extends well into the visible light regime. This result informs a material selection for low losses and broadband plasmonic response optoelectronic devices and photocatalytic reactors. Finally, the plasmon-driven hot carriers exhibit high anisotropy in momentum spaces, and it is mainly high-energy hot holes that are generated from the decay of SPP, which are instructional for designing applications that require carriers of specific momentum and energy and efficiently collecting hot holes in borophene monolayer. Moreover, the lifetime and mean free path of hot carriers reach 10 fs and 3 nm, respectively, which approach that of Au and Al compounds. As a result, we expect that the reported results will motivate further efforts to achieve low-loss broadband optical response and directional carrier transport in next-generation plasmonic optoelectronic devices and photocatalytic reactors.
